# Preoperative Evaluation of Adolescent Idiopathic Scoliosis: The Relevance of Routine Magnetic Resonance Imaging

**DOI:** 10.7759/cureus.86003

**Published:** 2025-06-14

**Authors:** Majdi M Abu Awida, Awni A Shyyab, Shaher A Aletan, Mohammad A Adamat, Heba O Al Ofieshat, Ashraf A Al-Tamimi, Monther M Alessa

**Affiliations:** 1 Neurology, Jordanian Royal Medical Services, Amman, JOR; 2 Internal Medicine, Jordanian Royal Medical Services, Amman, JOR; 3 Interventional Radiology, Jordanian Royal Medical Services, Amman, JOR; 4 Orthopedics, Jordanian Royal Medical Services, Amman, JOR

**Keywords:** adolescent idiopathic scoliosis, magnetic resonance image, neuraxial abnormities, preoperative, prevalence

## Abstract

Introduction

The evidence for using preoperative magnetic resonance imaging (MRI) to assess the whole spine in patients with adolescent idiopathic scoliosis (AIS) is not well established. However, it is routine to perform whole-spine MRI in patients with scoliosis who are scheduled to undergo surgical correction in our center to detect occult neural axis abnormalities that may not present with neurological symptoms but could influence surgical planning and safety.

Method

A retrospective single-center and descriptive evaluation of the medical records of AIS patients aged 10 to 18 who were admitted for surgical treatment at our institution between 2017 and 2022 to evaluate the value of the routine preoperative MRI. Descriptive statistics were utilized to analyze the data. Independent t-tests and chi-square tests (*X*²) or Fisher's exact tests were employed to compare groups as appropriate.

Results

Out of 106 patients evaluated, 10 patients (9.4%) were found to have neural axis abnormalities. Abnormal MRI findings were significantly more common in males than in females and were associated with increased thoracic kyphosis. However, there were no significant differences in the other parameters that were measured.

Conclusion

Even in the absence of neurological symptoms to identify any abnormalities of the neural axis, MRI may be useful in making decisions for the surgical treatment of AIS patients, particularly for those with risk factors such as male sex or exaggerated kyphosis.

## Introduction

Idiopathic scoliosis, the most prevalent type of scoliosis seen in adolescents, is present in those who are physically healthy, have no other medical conditions such as neurovascular or musculoskeletal congenital anomalies or tumors, and whose spines seem normal on a simple vertebral radiograph [1]. The origin of adolescent idiopathic scoliosis (AIS) remains unknown despite advances in treatment [2,3]. Given the idiopathic nature of the condition, it is crucial to pay careful attention to the physical examination, clinical history, and preferred radiological diagnosis method [4].

Advances in magnetic resonance imaging (MRI) have contributed to the diagnosis of neural axis abnormalities, including syringomyelia and Chiari malformations, in patients with suspected AIS in the absence of neurological signs [5-7]. However, ongoing debate surrounds the routine preoperative use of MRI in patients with putative idiopathic scoliosis reported in the literature [5,8,9] because it significantly raises healthcare costs and may only show minor variations from normal findings with no clinical significance [10].

All patients referred to the spinal deformity service at our institution get a whole-spine MRI as standard care in order to rule out any neural axis abnormality that may affect treatment or the consent procedure. In this study, we hypothesized that routine preoperative MRI in neurologically intact AIS patients could identify clinically relevant anomalies. Hence, the present study was conducted to investigate the prevalence of neural axis abnormalities in AIS patients and the clinical value of regular MRI investigations in the evaluation of patients with AIS prior to receiving surgical intervention.

## Materials and methods

A retrospective single-center and descriptive evaluation of the medical records of AIS patients aged 10 to 18 who were admitted for surgical treatment at King Hussein Medical City, Amman, Jordan, between 2017 and 2022 was conducted. The primary outcome was the prevalence of neural axis abnormalities detected on preoperative whole-spine MRI. Secondary outcomes included associations between abnormal MRI findings and demographic or radiographic parameters.

Patients with AIS requiring medical interventions who had a normal neurological examination and underwent a total spinal MRI were included in the study. Meanwhile, those with congenital, neuromuscular, or syndromic scoliosis and neurological deficits were excluded. The demographic and clinical data of the patients include age, sex, age at the onset of the disease, neurological findings, the presence or absence of neural axis abnormalities on MRI, shoulder height difference, coronal and sagittal balance, thoracic kyphosis, lumbar lordosis, and the type and magnitude of the curve.

The classification of patients was based on the Lenke et al. (2003) system [11]. The process of categorizing patients into one of the six main types. Furthermore, the patients were categorized based on their skeletal maturity using the Risser (1958) classification technique [12]. Coronal balance was clinically and radiographically classified as <10 mm, 10 to <20 mm, 20 to <30 mm, or ≥30 mm. The shoulder height difference was clinically and radiographically classified as <10 mm or ≥10 mm. Coronal parameters were measured as the distance between the C7 plumb line and the central sacral vertical line (CSVL). Sagittal balance was measured as the distance between the C7 plumb line and the posterosuperior corner of S1. Thoracic kyphosis was measured from the T5 upper endplate to the T12 lower endplate, and lumbar lordosis was measured from the L1 upper endplate to the S1 upper endplate via a lateral view. The Cobb method was used to determine the magnitudes of the curves. Cobb’s angle of ≤ 45° was considered mild scoliosis; between 46° and 80° denoted more moderate scoliosis; and more than 81° indicated severe scoliosis. Patients were divided into two groups: patients with and without neural axis abnormalities. All parameters were retrieved before correction surgery.

All patient data was handled with strict confidentiality, and data was analyzed anonymously by patient ID number. No contact was made with patients or their relatives. This study was approved by the Ethics Review Board at Royal Medical Services.

Analysis of the data was performed using IBM Corp. Released 2016. IBM SPSS Statistics for Windows, Version 25.0. Armonk, NY: IBM Corp. Continuous variables were summarized as means and standard deviations, while categorical variables were presented as frequencies and percentages. Independent t-tests were used to compare continuous variables between groups (with and without neural axis abnormalities). For categorical variables, the chi-square test (χ²) was used; Fisher’s exact test was applied when expected cell counts were below 5. A p-value of <0.05 was considered statistically significant.

## Results

**Figure 1:**
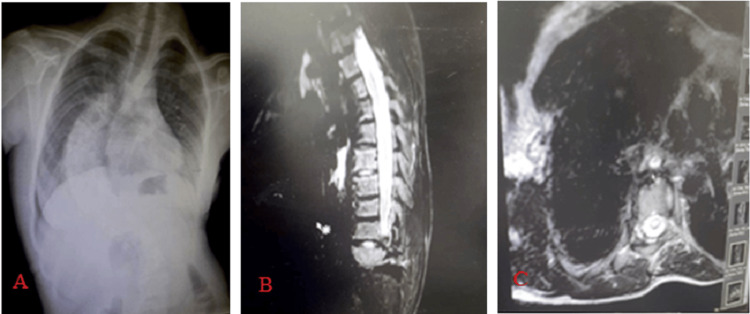
A fifteen-year-old female AIS patient with syrinx seen at C6–C7 on preparative MRI. A: Chest X-ray showing scoliosis, B: T2 sagittal (cervical and thoracic) showing C6-C7 syrinx, C: axial T2 C6-C7 syrinx AIS: Adolescent idiopathic scoliosis

**Figure 2:**
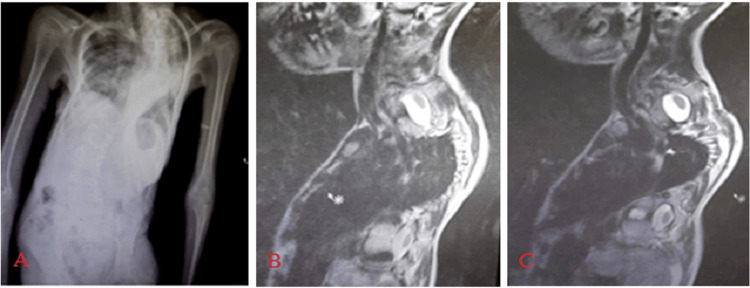
An 11-year-old male AIS patient with Dural ectasia seen at thoracic region with possible foraminal cyst seen left side of upper thoracic region measures about 15 mm on preoperative MRI. A: Chest X-ray showing scoliosis, B and C: sagittal MRI showing left lower cervical dural ectasia seen in the left upper thoracic region measuring 15 mm

The study evaluated a total of 106 AIS patients who were admitted for scoliosis surgery. All patients' physical and neurological exams were normal before surgery. Ten patients (9.4%) showed neural axis abnormalities as revealed in MRI before surgery. Magnetic resonance imaging revealed syringomyelia only in five patients, Arnold-Chiari malformation in two patients, tethered cord only in one patient, syringomyelia with tethered cord in one patient, and dural ectasia in one patient. All of those patients were referred to the neurosurgery department for further evaluation. Two patients out of six with syringomyelia only underwent Syringotomy; two patients with Arnold-Chiari malformation underwent posterior fossa decompression; one patient with a tethered cord only underwent tethered cord release; and one patient with syringomyelia with a tethered cord underwent tethered cord release. In terms of brain MRI, it was not performed as part of the routine protocol but was obtained when whole-spine MRI indicated possible craniovertebral junction abnormalities. A description of neural axis abnormalities detected on preoperative magnetic resonance imaging evaluation is shown in Table 1. Additionally, Figure 1 shows a 15-year-old female AIS patient with syrinx seen at C6-C7 on preoperative MRI, and Figure 2 shows an 11-year-old male AIS patient with dural ectasia seen at the thoracic region with possible foraminal cyst seen left side of upper thoracic region measures about 15 mm on preoperative MRI.

**Table 1:** Description of neural axis abnormalities detected on preoperative magnetic resonance imaging evaluation.

Type of neural axis abnormalities	No of cases	Neurosurgical intervention
Syrinx only	5	2
Arnold-Chiari malformation	2	2
Tethered cord only	1	1
Syrinx with tethered cord	1	1
Dural ectasia	1	0
Total	10	6

The average age of both groups was 13.9 years (SD = 2.3), and the average age at the time of diagnosis of both groups was 12.3 years (SD = 1.2). No significant difference between the groups was reported with regard to age or age at the time of diagnosis (p > 0.05). There were 35 male patients and 71 female patients. Most of the patients with neuroaxial abnormalities were male (70%) and there were significant sex-related differences across the groups (p = 0.014). A comparison between patients with and without neural axis abnormalities on preoperative MRI with regard to patient demographics is shown in Table 2.

**Table 2:** Demographic data and magnetic resonance imaging abnormalities (n=106 patients). * t-test, *** Fisher exact test, SD: Standard deviation

Variable	Total (n=106)	Normal group (n=96)	Abnormal group (n=10)	Test value	p-value
Age (years) Mean±SD	13.9±2.3	13.9±2.3	14.1±2.4	0.264^*^	0.790
Age at time of diagnosis (years) Mean±SD	12.3±1.2	12.2±1.6	12.4±1.7	0.283^*^	0.768
Sex, n (%)				6.82^ ***^	0.014
Male	35 (33)	28 (29.2)	7 (70)		
Female	71 (67)	68 (70.8)	3 (30)		

The thoracic kyphosis angle was significantly higher in patients with neural axis abnormalities than among those with normal findings (35.9 vs. 28.5, p = 0.002). No statistically significant differences were observed between patients with normal and those with abnormal MRI findings in terms of the other clinical and radiological parameters measured (p > 0.05). A comparison between patients with and without neural axis abnormalities on preoperative MRI with regard to clinical parameters is shown in Table 3.

**Table 3:** Clinical parameters and magnetic resonance imaging abnormalities (n=106 patients) * t-test, *** Fisher exact test, SD: Standard deviation

Variable	Total (n=106)	Normal group (n=96)	Abnormal group (n=10)	Test value	p-value
Curve magnitude (°) Mean±SD	58.2±12.8	57.7±13.2	62.2±7.5	1.04^*^	0.297
Thoracic kyphosis (°) Mean±SD	29.2±7.2	28.5±7.1	35.9±4.3	5.14^*^	0.002
lumbar lordosis (°) Mean±SD	46.7±5.7	46.6±5.7	47.1±3.1	0.251^*^	0.802
Coronal balance, n (%)				4.919^***^	0.131
<10 mm	52 (49.1)	44 (45.8)	8 (80)		
10 to <20 mm	33 (31.1)	32 (33.3)	1 (10)		
20 to <30 mm	14 (13.2)	14 (14.6)	0 (0)		
≥30 mm	7 (6.6)	6 (6.3)	1 (10)		
Shoulder-height difference, n (%)				1.37^***^	0.184
<10 mm	61 (57.5)	53 (55.2)	8 (80)		
≥10 mm	45 (42.5)	43 (44.8)	2 (20)		
Risser grade, n (%)				^8.18***^	0.080
Grade 0	5 (4.7)	4 (4.1)	1 (10)		
Grade 1	15 (14.2)	14 (14.6)	1 (10)		
Grade 2	23 (21.7)	23 (24)	0 (0)		
Grade 3	24 (22.6)	23 (24)	1 (10)		
Grade 4	17 (16)	13 (13.5)	4 (40)		
Grade 5	22 (20.8)	19 (19.8)	3 (30)		
Lenke classification, n (%)				^1.70***^	0.960
Main thoracic	43 (40.6)	38 (39.6)	5 (50)		
Double thoracic	17 (16)	16 (16.6)	1 (10)		
Double major	20 (18.9)	18 (18.8)	2 (20)		
Triple major	9 (8.5)	8 (8.3)	1 (10)		
Thoracolumbar	11 (10.4)	10 (10.4)	1 (10)		
Thoracolumbar with main thoracic	6 (5.7)	6 (6.3)	0 (0)		

## Discussion

In this retrospective single-center study, neural axis abnormalities were detected in 9.4% of adolescent idiopathic scoliosis (AIS) patients undergoing preoperative MRI despite normal neurological examinations. The most common abnormalities were syringomyelia (4.7%) and Arnold-Chiari malformation (1.9%). Male sex and increased thoracic kyphosis were significantly associated with abnormal MRI findings. No statistically significant differences were observed in age, curve magnitude, coronal or sagittal balance, shoulder height difference, lumbar lordosis, Risser grade, or Lenke classification.

These findings suggest that neural axis abnormalities may be present even in neurologically intact AIS patients and that male sex and hyperkyphosis could be considered potential risk indicators.

The prevalence of neural axis abnormalities in this study (9.4%) is consistent with previous literature. A systematic review reported a mean prevalence of 11.4%, with higher rates in patients with clinical risk factors (14.2%) compared to all AIS patients (10.5%) and preoperative populations (9%) [10]. Fruergaard et al. similarly reported an 8.9% prevalence, with syringomyelia being the most common finding (8.4%) [8].

In line with several previous studies [13-15], we found a significant association between increased thoracic kyphosis and neural axis abnormalities. Likewise, male sex has been reported as a risk factor in some studies [10, 16], although findings have not been universally consistent [4,17].

The absence of standard MRI screening guidelines for patients with idiopathic scoliosis has resulted in a broad range of indications for MRI screening among orthopedic surgeons. The literature suggests that MRI is indicated for various conditions such as pain, rapid progression, left thoracic deformity [17], neurologic disorder [10,17,18], early onset [10,17], double thoracic curvature [17] and male sex [10,17]. Contrary to that, those proposed indicators were not considered risk factors for neural axis abnormalities in the prospective study conducted by Fruergaard et al among 381 AIS patients [8]. Hence, the clinical significance of these indicators remains unclear. 

Anatomical changes in the spinal canal during and after surgery in patients with neural axis abnormalities [19,20] may increase their risk of having neurological complications [21]. Neurological complications were more common in patients with abnormalities (n = 368) than in patients without abnormalities (n = 3367) (0.82% vs. 0.18%) [10]. Although there are proponents of this view, Noordeen, Taylor, and Edgar (1994) have shown that neural axis anomalies are not linked to a higher rate of complications [7]. However, other experts believe that preoperative MRI is unnecessary in patients with idiopathic scoliosis unless they are experiencing neurological deficits such as a positive Babinski reflex and/or an exaggerated deep tendon reflex or pain [10,17,18,22]. Evidence suggests that an MRI is not required prior to correction of the spine, as demonstrated by a large series of 327 AIS patients with normal physical and neurological examinations [23].

In our study, all patients with neural axis abnormalities on MRI had spinal cord monitoring during surgery. Given the compression of the spinal cord resulting from the presence of a syrinx cavity, it is advisable to employ a burr rather than a hammer during facet joint removal in corrective surgery so as to minimize the risk of iatrogenic trauma to the spinal cord. The in-out-in (costovertebral placement) pedicle screw technique is a viable option to prevent spinal canal violations. In order to prevent the occurrence of iatrogenic trauma to the spinal cord, it is recommended that compressive maneuvers be executed on the convex side of the deformity instead of distractive maneuvers on the concave side of the deformity for the purpose of correction. A 15-year-old female AIS patient with syrinx seen at C6-C7 on preoperative MRI exhibited motor signal loss in the lower limbs during surgery, which postponed surgery and referred the patient to neurosurgical consultation for further investigation (Figure 1). 

Although routine MRI in AIS patients without neurological symptoms remains controversial, our findings indicate that a small but clinically relevant proportion of patients may harbor neural axis anomalies that could influence surgical planning and safety. In our cohort, 6 out of 10 patients with abnormal MRI findings required neurosurgical intervention.

Spinal cord monitoring was used in all patients, and a case of intraoperative neuromonitoring signal loss in a patient with syringomyelia highlights the potential surgical risk posed by unrecognized neural abnormalities. Such cases may benefit from modified surgical strategies, including more cautious instrumentation and correction techniques to minimize iatrogenic spinal cord injury.

Given that whole, spine MRI led to neurosurgical referrals and changes in surgical planning, selective preoperative MRI, especially in patients with thoracic hyperkyphosis or male sex-may be warranted.

This study has several limitations. First, its retrospective design introduces the risk of selection and information bias. Second, the sample size, particularly the number of patients with neural axis abnormalities, is relatively small, which may limit the generalizability of the findings. Third, brain MRI was not routinely performed, and its selective use may underestimate associated cranial abnormalities. In addition, we did not assess other factors such as curve direction, progression rate, or back pain, which have been suggested as possible MRI screening criteria. Finally, cost-effectiveness was not evaluated, and no long-term outcomes were analyzed.

While our findings suggest that preoperative MRI may detect clinically significant abnormalities in a subset of AIS patients without neurological symptoms, the evidence from a retrospective, single-center cohort is not sufficient to recommend universal MRI screening. A risk-based approach, informed by patient characteristics such as thoracic hyperkyphosis and male sex, may offer a more cost-effective strategy.

Future large-scale, prospective studies are needed to validate these risk indicators and to define clear, evidence-based guidelines for MRI use in the preoperative evaluation of AIS.

## Conclusions

Neural axis abnormalities were detected in 9.4% of patients with adolescent idiopathic scoliosis (AIS) in this cohort. Male sex and increased thoracic kyphosis were significantly associated with these abnormalities, while other clinical and radiographic parameters showed no significant differences. Although all patients in this study were neurologically asymptomatic, MRI identified potentially important findings that could influence surgical planning. These results suggest that preoperative MRI may have value in selected AIS patients, particularly those with risk factors such as male sex or exaggerated kyphosis. Given the limitations of this retrospective, single-center study, we do not recommend routine MRI for all AIS patients. Instead, our findings support the need for further research into cost-effective, risk-based screening strategies to guide preoperative imaging decisions.
